# Impact of organization decision making styles and safety accountability on occupational health and safety implementation: The moderating role of mimetic motives

**DOI:** 10.3389/fpubh.2022.1004767

**Published:** 2022-11-14

**Authors:** Muhammad Aamir Nadeem, Lilis Surienty, Md. Mahmudul Haque

**Affiliations:** ^1^School of Management, Universiti Sains Malaysia, Penang, Malaysia; ^2^Labuan Faculty of International Finance, Universiti Malaysia Sabah, Kota Kinabalu, Malaysia

**Keywords:** occupational health and safety implementation, safety accountability, organizational decision-making styles, agriculture, mimetic motives

## Abstract

The agriculture sector is a traditional economic pillar of many emerging economies. However, it is facing greater occupational health and safety (OHS) challenges in Pakistan, and its performance is continuously decreasing. An effective OHS implementation provides better control over OHS challenges and may help to restore its former glory. Therefore, this study aims to explore different organizational decision-making styles and safety accountability to put OHS into practice in this sector. Based on institutional theory, a theoretical framework was developed. Two hundred and eighty-seven agriculture farms in Punjab, Pakistan were surveyed and analyzed using SmartPLS 3.3.7. The findings revealed that implementation styles (rational and incremental) and safety accountability positively impact OHS implementation. Similarly, the moderating role of mimetic motives was found positively significant in the relationship between rational style and OHS implementation, and negatively significant in the relationship between incremental style and OHS implementation. While no moderating effect of mimetic motive was found between safety accountability and OHS implementation. This study suggested that OHS implementation should not be viewed as a social or technical issue alone. Strategic arrangements should be made at the organizational level to gain better control over OHS challenges by considering the institutional environment in which the organization operates.

## Introduction

The OHS implementation at the organizational level in the 21st century has long been a cause of concern. According to International Labor Organization (ILO), OHS means the science of anticipation, recognition, evaluation, and control of hazards arising in or from the workplace that could impair the health and well-being of workers, considering the possible impact on the surrounding communities and general environment (1). Based on the definition, it is the prime responsibility of any organization to provide a safe working environment and consider the health and well-being of all stakeholders. In the OHS domain, work-related diseases and injuries are the major occupational health problem (2, 3). The report by Workplace Safety and Health Institute of 2017 revealed that approx. 2.78 million deaths occur annually across the world (2). It further highlighted that 86.3% of the total deaths were attributed to occupational diseases and 13.7% to fatal accidents. Based on ILO (4) assessments, more than 2.3 million workers die each year from work-related diseases and injuries, 160 million suffer from non-fatal work-related diseases, and 313 million from non-fatal injuries. Therefore, concerns about OHS are widespread across both industrial and emerging economies (5, 6). For instance, in Great Britain, 123 workers died per 1,000 in work-related accidents in 2021-22 (7). Additionally, the report found that construction and agriculture have the highest rates of work-related accidents. Similarly, more than 1,700 work-related accidents were reported until March 2022 in Malaysia, while agriculture was the second most affected sector after manufacturing (8). In Pakistan, agriculture is a prominent sector for worker-related injuries and deaths. This sector accounted for 29.25% of workers' deaths in 2020-21 (9). Consequently, work-related injuries and deaths implicate an imminent cost on the socioeconomic systems, destabilizes the worker earning capacity, and adversely affect the nation's productivity level (10). According to the recent estimates of ILO (4), more than 4% of the world GDP per year is lost as a result of work-related diseases and injuries which rises to 6% in emerging economies.

In recent decades, the OHS domain has gained popularity both in the industrial and scholarly world. The OHS implementation has directly supported the Sustainable Development Goals (SDGs), like SDGs 3.9, 8.8, and 16.6 (11). Ivascu et al. (12) argued that OHS facilitates organizations to achieve sustainability and innovation performance. Fonseca and Carvalho (13) stated that organizations having OHS certification performed better to achieve sustainable development goals. Additionally, Zorzenon et al. (14) posited that OHS and digital technologies help to promote SDGs. Ávila-Gutiérrez et al. (15) claimed that OHS must be aligned with current industry norms to achieve the SDGs and smooth transition from Industry 4.0 to 5.0.

A considerable number of study has been published on occupational health and safety. These studies are more focused on oil and gas (16), transportation (17), manufacturing (18), and mining (19). However, agriculture is the most neglected sector in terms of OHS implementation. For example, there is a lack of empirical evidence in agriculture, especially in agro-based economies like Pakistan. Although few researchers attempted to perform empirical research in agriculture or related sectors in Pakistan (10, 20, 21). However, the prior studies contextualized OHS as a human indicator like gender (21) and occupations (16). Noman et al. (10) argued that organizational and human perspectives must be examined separately to understand the OHS setups in developing countries better. Based on organizational context, a decision strategy is considered a vital factor in promoting OHS implementation. Andrews et al. (22) argued that organizational decision-making styles (rational & incremental) are vital to achieving OHS implementation. Likewise, Otok et al. (23) claimed that organizational decision-making styles help to mitigate disaster risk in developing countries. Additionally, in the era of COVID-19, safety accountability is a prominent enabling factor for OHS implementation at the organizational level (24). Although the OHS research domain has gained significant attention, several lines of inquiry need urgent action, like organizational strategic decision-making style (22, 23), and safety accountability (24). Additionally, Ju et al. (25) claimed that some external stimuli or motives influence a firm's strategic actions to promote OHS implementation. Generally, these motives are known as mimetic motives. Taylor and Buumba (26) argued that mimetic motives affect the OHS implementation practices in the service sector. They further argued that more research is required in other sectors of the economy.

Despite being a vital and integral aspect of organizational objectives, OHS implementation has rarely been studied. Notably, there is a lack of research that elucidates OHS implementation in the agricultural sector. Therefore, the present study aspires to investigate the impact of different organizational decision-making styles, safety accountability, and mimetic motives in OHS implementation by raising the research question that “how different types of organizational strategies impact OHS implementation?”. To address the research question, this study will be able (a) to evaluate the impact of organizational decision-making styles & safety accountability on OHS implementation and (b) to examine the moderating effect of mimetic motives on decision-making styles and OHS implementation. A number of contributions and implications will result from this effort once the research objectives have been achieved. For example, this study will enhance the theoretical understanding of institutional theory from an OHS implementation standpoint. Similarly, it will help managers to design OHS implementation programs, especially in the agriculture sector. Furthermore, the research findings may help national authorities to formulate policies regarding training and technology selection for OHS implementation. Most importantly, this study will provide the liberty to agriculture organizations to choose implementation style for OHS implementation as per their competencies, environmental uncertainties, and their desire to be like other organizations.

The following section presents the theoretical framework and hypothesis development. After that, material and methods, results and discussion of the findings are presented. Finally, the conclusion, limitations, and future research avenues are discussed.

## Theoretical foundation and hypotheses development

### Institutional theory and OHS implementation

Institutional theory refers to managing an organization's environment. Phillip Selznick initially defined its fundamental concept in the year 1949. Subsequently, John Meyer and Brian Rowan, as well as Paul J. DiMaggio and Walter W. Powell advanced it in 1977 and 1983, respectively (27). This theory not only explains how certain institutions influence the organization's behavior and decision-making process in which it operates but also why organizational structures and practices change (28, 29). Six key concepts make the basis of this theory: legitimacy, isomorphism, rational myths, loose coupling, diffusion, and the infusion of value (27). Collectively, these concepts ensure the survival of an organization in the institutional environment with both technical (i.e., capital and labor) and social (i.e., legitimacy and status) perspectives. Therefore, legitimacy and isomorphism are considered central assumptions of this theory.

As an essential organizational component, OHS implementation is seen as a social and technical aspect of managing an organization's environment. It reflects how an organization promotes health and safety at the workplace, which impacts several dimensions of performance, such as productivity, absenteeism, and employee satisfaction (30). Jilcha et al. (31) stated that implementation of the OHS requirements is not the only responsibility of internal stakeholders, but external stakeholders such as governing bodies, unions, insurance agencies, and other higher institutions are also equally responsible. For this reason, the institutional theory could result in better OHS implementation due to the effective involvement of both internal and external stakeholders.

Furthermore, various past research work [e.g., (32–38)] in occupational health and safety have employed this theoretical ground to understand the organizational behavior in responding to the OHS issues. The other strength of this theory is that it supports organizational transformation, even if the technical or economic advantages are lacking (39, 40). Therefore, this is also the reason that the present study employs the institutional theory to explain the influence of different institutions (i.e., factors) on OHS implementation.

### Implementation style and OHS implementation

Implementation style is a process of putting a plan into practice. Pollitt and Bouckaert (41) emphasized that understanding the implementation dynamics of a program requires an awareness of implementation styles. Similarly, Andrews et al. (22) stated that the successful execution of a plan or program depends on a particular implementation style, which, as a result, has substantial significance for the performance of an organization. Several styles of implementation exist in the literature. However, researchers explicit them into two main categories: rational style and incremental style (22, 42). The rational style is a systematic and well-planned approach that prioritizes organizational changes and gets people to follow the precise procedure to adopt these changes. Whereas incremental style is a fluid nature style of organizational change which encourages modification step by step based on the ground realities (22). Past studies revealed mixed findings on implementation styles. For example, Andrews et al. (43) used these implementation styles to check service performance in public organizations. The study revealed that no implementation style by itself is effective, but that style is contingent on organizational strategic orientation. Similarly, Andrews et al. (22) studied the individual and combined effect of rational and incremental implementation styles in public sector organizations in Turkey. It found that the organization performs better that use a combined implementation style more than those that emphasize a single style. Whereas the absence of a style may lead to worse performance. On the contrary, Balogun and Jenkins (44) explain that the rational style gives better results in reactive situations or crises. In contrast, the incremental style best suits managers to adopt in proactive situations. As the OHS program not only provides controls in proactive ways but also enables organizations to handle hazardous situations by remedial measures. Therefore, analyzing both implementation styles is important to assess which style leads to better OHS implementation. Thus, the following hypotheses are proposed:

H1: The rational style has a significant positive impact on OHS implementation but is greater than the incremental style.
H2: The incremental style has a significant positive impact on OHS implementation but less than the rational style.

### Safety accountability and OHS implementation

Safety accountability is essential for OHS implementation. It creates and establishes in each employee a sense of obligation to perform assigned tasks effectively and efficiently. For example, management is responsible to design policies and procedures, clarify roles and responsibilities, provide appropriate training and infrastructure, and granting employees sufficient authority to do their part in OHS implementation well (45). Similarly, it is the responsibility of safety personnel to encourage management and all other employees to OHS implementation. It is because OHS implementation is an organization-wide effort. The safety department or safety personnel cannot ensure its success on their own alone. Therefore, everyone in the organization is responsible for its success (46). However, without safety accountability, a safety program implementation is likely to be unsuccessful or only ceremonial. Kim et al. (46) argued that an organization cannot achieve safety excellence until its management and all other employees are not accountable for their measurable responsibilities. Moreover, Mlynek (47) stated that safety accountability is not a punitive strategy but rather a proactive means of fostering a culture of responsibility. As such, the following hypothesis was developed:

H3: Safety accountability has a significant positive impact on OHS implementation.

### Moderating role of mimetic motives and OHS implementation

Mimetic motives refer to the extent to which an organization implements a program by mimicking the best practices of leading organizations in an attempt to gain legitimacy. This trend is most common in developing countries because it saves both cost and time (48). Moreover, Ansari et al. (49) argued that mimicking behavior consist of two perspectives; laws & regulations, and societal norms & values. The first perspective stems at to achieve legitimacy, which is vital in securing resources for continued existence over the long run (29). Whereas the second perspective is of moral origin, which emboldens ethical and responsible behavior regardless of the outcomes (28, 50).

McBain-Rigg et al. (51) identified peer-to-peer networking, industry representatives, government, and media as the most influential agents for safety implementation in the agriculture and fishery sector of Australia. Similarly, Iatridis et al. (52) found that governmental authorities, NGOs, and the local community are mimetic motives that influence them to commit fully to certify management standards, such as OHSAS 18001. Another perspective proposed by DiMaggio and Powell (39), is that mimetic motives are especially relevant in those organizations where technology is poorly understood, the environment is uncertain, and goals are ambiguous. Thus, based on institutional theory, this study understands mimetic motives as institutional motives that push organizations to adopt OHS implementation as strategy implementation. Furthermore, this study expects that the organizations that have a strong influence from mimetic motives, their decision-making styles for implementation, and safety accountability would be high for OHS implementation. This indicates moderating effects of mimetic motives on the relationships between exogenous and endogenous variables. Therefore, it is necessary to explore the role of mimetic motives in implementing the OHS. Thus, the following hypotheses were developed.

H4: Mimetic motives significantly moderate the relationship between rational style and OHS implementation.
H5: Mimetic motives significantly moderate the relationship between incremental style and OHS implementation.
H6: Mimetic motives significantly moderate the relationship between safety accountability and OHS implementation.

## Materials and methods

### Study area and population

This study focused on the province of Punjab (31.1704° N, 72.7097° E). It is most prominent in agriculture production and has the most significant share in the national economy compared to other provinces. Therefore, the targeted population was all agricultural farms in this province. The latest provincial government statistical record showed that there are 5,249,800 agriculture farms in the whole province (53). Although all districts in the province of Punjab are notable for different varieties of agricultural products. However, the district Bahawalnagar, Bahawalpur, Bhakkar, Sargodha, Multan, Pakpattan, Okara, Jhang, and Layyah were chosen purposively as the study area because these districts contain large farms, and it is assumed that large agriculture farms have dedicated safety personnel and resources to cope with OHS issues.

### Unit of analysis

The agriculture farm was chosen as the unit of analysis because this study is aimed at providing a holistic view of OHS implementation in the agriculture sector of Pakistan. Moreover, the respondents of this study participants were safety person-in-charge (i.e., owners, OHS managers, OHS officers, or OHS representatives). These individuals were selected as respondents for three main reasons. First, they are linked with all activities of other departments and speak for OHS at the executive level. Second, they have sufficient practical and professional knowledge to implement the latest OHS programs. Third, these people provide reliable information due to their key role in OHS implementation and continuous monitoring.

### Sample size and sampling technique

The G^*^Power software of version 3.1 was used to calculate the minimum sample size, as recommended by Joseph et al. (54) and Memon et al. (55). A sample size of 153 was calculated with the settings of statistical power of 95%, probability error of 5%, and with a medium effect size of 0.15 for four predictors and three interaction terms. The agricultural farms were selected through purposive sampling. This technique was applied because a comprehensive and up-to-date list of agriculture farms was not readily available. Kumar (56) described that the purposive sampling technique is more suitable, where a complete list of the total population is unavailable to researchers.

### Measures and data collection producer

The questionnaire was divided into two sections. The first section contains the demographics of the respondents, such as gender, age, qualification, designation, working experience, nature of business, farm type, and number of employees. Whereas the second section includes the measures of study constructs. The measures of both rational style (5-items) and incremental style (5-items) were adapted from Andrews et al. (22). The items of safety accountability were adapted from Molenaar et al. (57), which contains four items in total. Similarly, the three items for measuring mimetic motives were adapted from Hillebrand et al. (58). Finally, the intention for OHS implementation was measured with four items adapted from Hossain et al. (59). Moreover, the data of the second section was collected on a 7-point Likert scale from 1 (“strongly disagree”) to 7 (“strongly agree”). Keeping the education level of respondents in mind, the questionnaire was translated into Urdu and sent to one academic person and two OHS professionals (i.e., OSH consultants) for content validity. After their feedback, a pre-test with 37 actual respondents was also conducted to confirm the comprehension and reliability of the questionnaire. No major amendments surfaced, aside from a slight change in wording. Moving forward, a paper-based questionnaire was personally handed over to respondents. The questionnaire distribution with this method was aimed at generating a higher response rate (60). A cover letter, stating the confidentiality of data and the nature of the study, was also attached to each questionnaire. A total of 400 farms were targeted through different channels. Moreover, data was collected in a single sitting, specifically from January 2022 to April 2022.

### Data analysis

**Figure 1:**
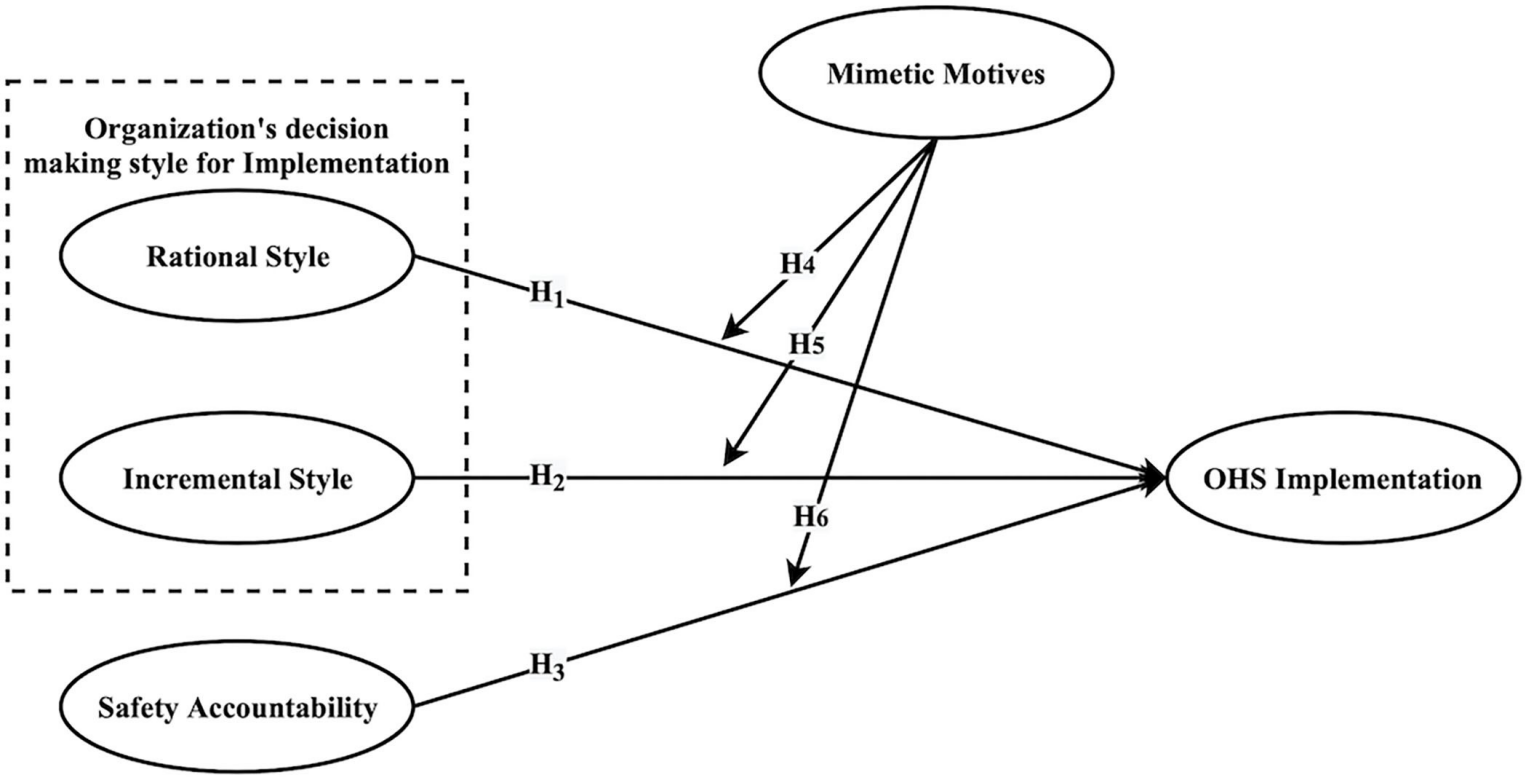
Research framework.

The PLS-SEM technique was adopted to examine the theory-based conceptual model that is specified in Figure 1. More importantly, this technique provides robustness in analysis and does not rely on data distributional assumptions (54). Two steps are strictly followed. In the first step, the reliability and validity of constructs were assessed through outer loadings, composite reliability (CR), and average variance extracted (AVE). HTMT was also examined to confirm the establishment of discriminant validity. This is then followed by structural model estimation. This step includes the assessment of the coefficient of determination (R^2^), path coefficient (β), effect size (f^2^), predictive relevance (Q^2^), model fit, and PLS prediction. Furthermore, a bootstrapping procedure with 5,000 iterations was used to test the significance of hypothesized relationships. Besides, moderation was evaluated using an orthogonalization approach (54). Data were analyzed using SmartPLS of version 3.3.7 (61) and SPSS of version 25.

## Results

### Demographics Findings

Most of the participants were men (97.91%) with an age bracket of 31–40 years (47.04%) and had a bachelor's qualification (72.82%). Those who responded as safety in-charge persons (i.e., safety manager, safety officer) accounted for 47.73%, while those who responded as safety representatives or owners were 52.27%. The majority of farms had employees below 50 (79.10%) and livestock nature of business (41.11%). Moreover, private agriculture farms participated in this survey the majority (95.47%). The detailed findings of demographics are illustrated in Table 1.

**Table 1:** Demographic findings.

**Criteria**	**Description (*n* = 287)**	**Numbers**	**Percentage (%)**
Gender	Male	281	97.91
	Female	06	2.09
Age (Years)	≤ 20 years	02	0.70
	21 to 30 years	61	21.25
	31 to 40 years	135	47.04
	41 to 50 years	75	26.13
	Above 50 years	14	4.88
Respondent's highest qualification	Secondary school certificate	08	2.79
	Bachelor degree	209	72.82
	Diploma	20	6.97
	Postgraduate degree	50	17.42
Current position	Owner	32	11.15
	Safety manager	23	8.01
	Safety officer	114	39.72
	Safety representative	118	41.12
Employee's strength	Below 10 employees	10	3.49
	11 to 50 employees	217	75.61
	51 to 100 employees	48	16.72
	Above 100 employees	12	4.18
Business nature	Crops	37	12.89
	Livestock	118	41.11
	Forestry	22	7.67
	Fishery	24	8.36
	Integrated	86	29.97
Type of farm	Public	13	4.53
	Private	274	95.47

### Common Method Variance (CMV)

The data on all study constructs were collected in a single sitting and from the same respondent, common method bias could be a potential problem. Three remedies were used to alleviate this problem. First, the participants were informed that there were no wrong or correct answers and that their responses would be anonymous and would not use for their performance evaluation. Additionally, the readability and comprehension of all the items were improved by keeping the question specific and concise by avoiding ambiguous terms (62). Second, Harman's Single Factor test was carried out to check the common method bias in the data. In this approach, all eighteen items were loaded to a single factor using principal component analysis with the varimax rotation method. The single factor explained variance of 28.271%, far below the 50% threshold. Third, a full collinearity test for both endogenous and exogenous variables was applied to examine this problem. The pathological (inner) VIF for all constructs ranged from 1.091 to 1.121, which is less than 3.30 (63), confirming again that CMV is an unlikely threat to this study.

### Measurement model assessment

The framework of the current study contained three exogenous, one endogenous, and one moderating construct. All were proposed as first-order reflective constructs. Therefore, the recommendations of Joseph et al. (54) were followed for measurement model assessment. Convergent validity and internal consistency reliability were assessed in the first step, while discernment validity was assessed in the later step. For convergent validity, the factor loading of all items must be higher than 0.703, and the average variance extracted (AVE) should be equal to or higher than the threshold of 0.500 for each scale. Whereas Cronbach's alpha and the composite reliability (CR) of all the reflective constructs must exceed 0.700 to show internal consistency reliability (54). The findings are tabulated in Table 2. It revealed that Cronbach's alpha, CR, and AVE values met the threshold criteria. Similarly, the factor loadings of all indicators, excluding RS5, were also found to be above 0.703. However, RS5 was retained due to three reasons. First, this indicator was vital and of absolute importance for the study. Second, the value of factor loading was very close to the threshold. Third, the AVE value of the relevant construct was meeting the threshold criteria without excluding it. Thus, the constructs of the proposed model were found to have satisfactory convergent validity and internal consistency reliability.

**Table 2:** Measurement model: VIF, internal consistency reliability and convergent validity.

**Constructs**	**Indicator**	**VIF**	**Factor loading**	**Cronbach's alpha**	**Composite reliability**	**Average variance extracted (AVE)**
Rational style	RS1	1.443	0.733	0.798	0.860	0.553
	RS2	1.516	0.736			
	RS3	1.631	0.786			
	RS4	1.669	0.766			
	RS5	1.464	0.692			
Incremental style	IS1	2.689	0.872	0.909	0.932	0.733
	IS2	2.543	0.860			
	IS3	2.300	0.839			
	IS4	2.402	0.851			
	IS5	2.459	0.858			
Safety accountability	SA1	1.965	0.837	0.831	0.887	0.663
	SA2	1.864	0.826			
	SA3	1.682	0.800			
	SA4	1.646	0.795			
Mimetic motives	MM1	1.829	0.794	0.797	0.867	0.687
	MM2	1.680	0.753			
	MM3	1.630	0.929			
OHS implementation	OHS1	1.917	0.837	0.867	0.909	0.715
	OHS2	2.491	0.869			
	OHS3	2.388	0.862			
	OHS4	1.842	0.813			

Henseler et al. (64) suggest that the Heterotrait-monotrait (HTMT) ratio of correlation should be tested for discriminant validity. It reflects the distinction of the framework's constructs (63, 64). According to recent literature, the HTMT ratio is more precise and preferred than the Fornell and Larcker criterion and cross-loading method (54). The maximum threshold value of 0.85 indicates sufficient discriminant validity of the constructs (65). The results presented in Table 3 demonstrate that HTMT < 0.85, meaning that the study's constructs are distinct. Hence, sufficient discriminant validity exists.

**Table 3:** Measurement model: Discriminant validity (HTMT_0.85_).

**Construct**	**Mean (SD)**	**Rational style**	**Incremental style**	**Safety accountability**	**Mimetic motives**	**OHS implementation**
Rational style	5.617 (1.116)	-				
Incremental style	5.216 (1.382)	0.278	-			
Safety accountability	5.536 (1.174)	0.311	0.281	-		
Mimetic motives	4.148 (1.407)	0.099	0.074	0.151	-	
OHS implementation	6.010 (1.010)	0.524	0.507	0.323	0.09	-

Moreover, multicollinearity was also checked through the outer variance inflation factor (VIF) of all items before testing the structural model. Joseph et al. (54) recommend that checking VIF reduces the biasness in the hypothesized relationships. The values of outer VIF ranged from 1.443 to 2.689 (see Table 2), which were far below 3.3 (63), showing no multicollinearity problem in the data.

### Structural model assessment

Following that, the model's path quality, exploratory power model, and strength of the proposed hypotheses were examined in the structural model assessment. The path quality was estimated through PLS blindfolding procedure using the Q^2^ value. The Q^2^ value greater than 0 shows a predictive accuracy of the model (65). Additionally, the Q^2^ value of 0, 0.25, and 0.50 represents small, medium, and large predictive relevance of the path model. Table 4 revealed a Q^2^ value of 0.260 which means that the model had a medium-size predictive accuracy.

**Table 4:** Structural model: Path quality and exploratory power.

**Construct**	**Path quality of the model**		**Exploratory power of the model**			
	**Q^2^**	**Decision**	**R^2^ without moderator**	**Decision**	**R^2^ with moderator**	**Decision**
OHS Implementation	0.260	Medium	0.333	Medium	0.393	Substantial

The exploratory power of the model was estimated using coefficients of determination (R^2^) of the endogenous construct (i.e., OHS implementation). The R^2^ value tells the total variance explained in the endogenous construct by exogenous constructs (66). The R^2^ value for OHS implementation indicated a medium exploratory power without a moderator and substantial with a moderator (see Table 4). It means that mimetic motives as a moderator increase the 6% exploratory power of OHS implementation, which is accounted for by the organization's decision-making style for implementation (i.e., rational and incremental) and safety accountability. Next, the strength and statistical significance of the proposed hypothesized relationships were tested. For this purpose, *t*-statistics and path coefficients (β) were evaluated using a bootstrapping procedure with 5,000 subsamples (63). Table 5 revealed that rational style (H1: β = 0.353, t = 6.069, *p* < 0.001), incremental style (H2: β = 0.319, t = 5.085, *p* < 0.001), and safety accountability (H3: β = 0.120, t = 2.102, *p* < 0.05) are having positive significant impact on OHS implementation. Thus, all three direct hypotheses were supported.

**Table 5:** Structural model: Hypothesized relationship testing and effect size.

**Relationships**	**β**	**SD**	***t*-values**	***p*-values**	**Decision**	***f^2^* statistics**	**Effect size**
H1: Rational Style → OHS Implementation	0.353	0.058	6.069	< 0.001	Accept	0.182	Medium
H2: Incremental Style → OHS Implementation	0.319	0.063	5.085	< 0.001	Accept	0.150	Medium
H3: Safety Accountability → OHS Implementation	0.120	0.057	2.102	0.018	Accept	0.021	Weak
H4: RS*MM → OHS Implementation	0.138	0.065	2.113	0.018	Accept	0.024	Weak
H5: IS*MM → OHS Implementation	−0.170	0.075	2.275	0.012	Accept	0.043	Weak
H6: SA*MM → OHS Implementation	0.094	0.137	0.682	0.248	Reject	0.012	Weak

RS, Rational Style; IS, Incremental Style; SA, Safety Accountability; MM, Mimetic Motives; OHS, Occupational Health and Safety; β, Path Coefficient; S.D, Standard Deviation.

**Figure 2:**
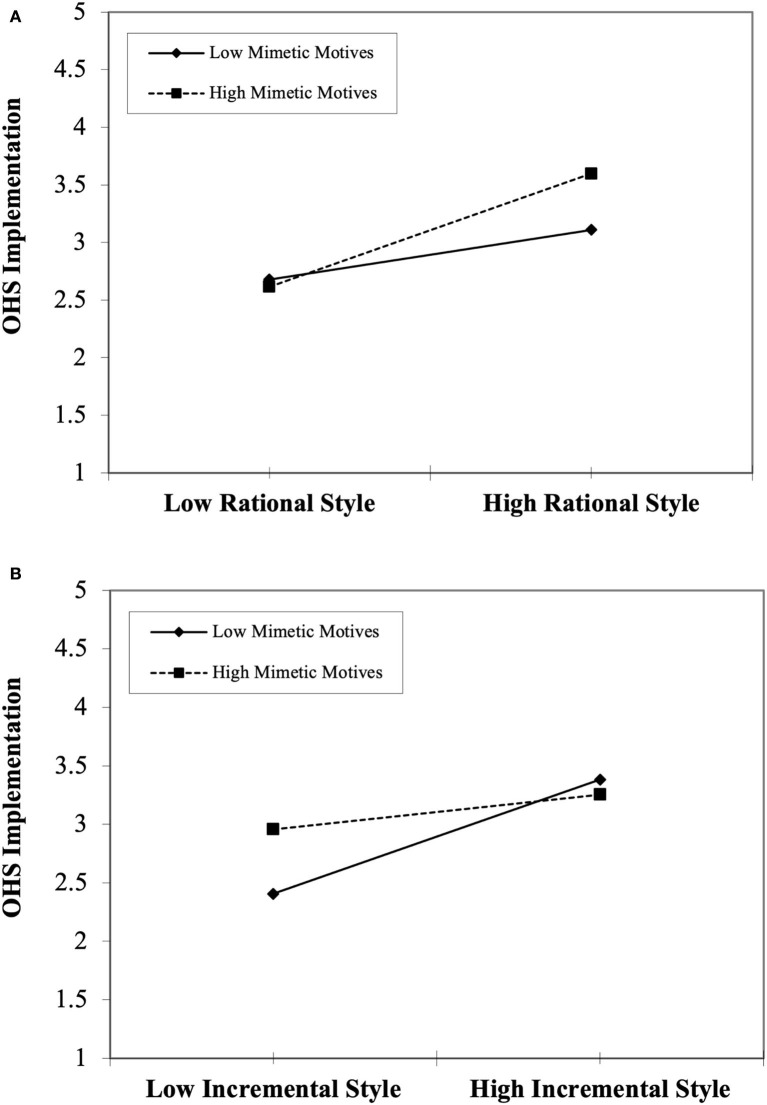
**(A)** Interaction of rational style and mimetic motives on OHS implementation. **(B)** Interaction of incremental style and mimetic motives on OHS implementation.

Regarding moderation, the interaction terms were created in SmartPLS and analyzed using an orthogonalization approach. Joseph et al. (54) argued that the orthogonalization approach is an extension of the product indicator approach and gave better results than other approaches (i.e., two-stage, and product indicator). The results tabulated in Table 6 showed that mimetic motives (H4: β = 0.138, t = 2.113, *p* < 0.05) positively influenced the relationship between rational style and OHS implementation. Whereas mimetic motives (H5: β = −0.170, t = 2.275, *p* < 0.05) negatively moderate the relationship between incremental style and OHS implementation. However, contrary to hypothesized relationship, mimetic motives (H6: β = 0.094, t = 0.682, *p* > 0.05) were not found to have a moderating effect on the relationship between safety accountability and OHS implementation in either a positive or negative direction and hence rejected. Thus, two moderating hypotheses were found to be supported, and one was rejected. In addition, moderating effect of mimetic motives was also analyzed using a simple slop test. Figure 2A shows that the interaction effect of mimetic motives is consistent with the prediction of H4. The upward increasing slope showed that rational style and OHS implementation are positive for both low and high mimetic motives. However, OHS implementation is higher when there is a low rational style but for those with low mimetic motives. On the contrary, OHS implementation increased through rational style increased for those agriculture farms with higher mimetic motives. Similarly, Figure 2B shows that when the involvement of mimetic motives is low, the impact of incremental style is higher on OHS implementation. However, when mimetic motives are high, then the relationship between incremental style and OHS implementation is weakened.

**Table 6:** Structural model: Pls predict.

**Construct**	**Indicators**	**RMSE (PLS-SEM)**	**RMSE (LM)**	**Difference**	**Q^2^_predict**
OHS implementation	OHS1	1.292	1.494	0.202	0.266
	OHS2	0.813	0.941	0.128	0.234
	OHS3	0.852	1.001	0.149	0.184
	OHS4	1.277	1.465	0.188	0.187

More to this, effect size (f^2^) was also calculated based on the recommendation of Chin (67). It measures the strength of a specific exogenous construct on an endogenous construct by mean change in the coefficient of determination (R^2^). Cohen (66) described the threshold of 0.35, 0.15, and 0.02 for substantial, medium, and small effect sizes. The statistics in Table 5 are indicative of small to medium effect size.

Likewise, the goodness of the model was also assessed in this study. A long list of fit indices such as standardized root-mean-square residuals (SRMR), unweighted least square discrepancy (d_ULS), geodesic discrepancy (d_G), Normed Fit Index (NFI), RMSEA, and CFI have been proposed in the literature. However, the standardized root-mean-square residuals (SRMR) index proposed by Hu and Bentler (68) is dominant as an approximate model fit criterion in the PLS-SEM context (69). Hu and Bentler (68) suggested a cut-off value of SRMR ≤ 0.08 for a good fit. The SRMR = 0.055 was estimated for this study, well below the threshold, indicating a good fit for the proposed model. It is worth mentioning that the absolute application of any fit measure remains not fully developed, and GoF assessment is unnecessary in general in PLS-SEM (65).

In the end, PLS Predict was used to check the out-of-sample predictive relevance (power) of the model with a default setting of k = 10 (70). As Q${}_{\text{Predict}}^{2}$ > 0 and the prediction errors in this study are highly symmetrically distributed, a comparison of RMSE values of PLS-SEM and linear regression model (LM) was held as per the recommendation of Shmueli et al. (70). The results in Table 6 show that all indicators of OHS implementation hold true for PLS-SEM < LM, which confirmed the medium out-of-sample predictive power of the model.

## Discussion

The agriculture sector deals with different occupational accidents, and consequently the need for increased intention of OHS implementation. The farm managers and workers are the ones who put OHS implementation into practice. As the implementation styles and safety accountability influence the OHS implementation, the relationship between the interaction of organizational decision-making styles (rational and incremental), safety accountability, and mimetic motives on OHS implementation were investigated. The findings revealed that mimetic motives, in combination with a rational style, had a positive and significant influence on OHS implementation. The interaction of mimetic motives and incremental style, on the other hand, has a negative but significant effect on OHS implementation. However, there was no substantial influence on OHS implementation when mimetic motives were coupled with safety accountability.

The positive impact of both rational and incremental styles on OHS implementation was supported. This is in line with the findings of Andrews et al. (22) who showed that incremental style is positively associated with a higher level of effectiveness but less than rational style. These findings imply that the agriculture sector may minimize occupational injuries and diseases by implementing the OHS program either in a rational or incremental fashion. However, it is worth mentioning that the rational style for OHS program implementation was sub-optimal to the incremental style in the context of the Pakistani agriculture sector.

Similarly, the interaction effect of mimetic motives with implementation styles (rational and incremental) on OHS implementation was also supported. However, the results were surprising. The interaction of mimetic motives with rational styles increased the impact of OHS implementation, while interaction with incremental style decreased the impact of OHS implementation. Taylor and Buumba (26) argued that a particular style typically depends on unforeseen circumstances, such as leadership vision, organizational climate, priorities, and resource availability. Moreover, it might be possible that current management would take pressure from trendsetters, business media, and their competitors to embrace OHS. Therefore, in the presence of these pressures, rational style along with mimetic motives may give better interactional results and may make it a better choice for OHS implementation. However, it might be concerning because such OHS implementation is usually ceremonial or superficial. On the contrary, Cândido and Santos (71) revealed that the implementation process requires a longer time for the successful execution of a program. Therefore, a number of factors such as change of leadership, and governance system of regulatory authorities might influence the OHS implementation. Given that implementation style may likely change with the change of management during the implementation phase of the OHS program or trend-setter's fashion become old or obsolete. Therefore, the interaction of incremental style and mimetic motives might result in a negative trend in OHS implementation.

Furthermore, the result showed a positive and significant impact of safety accountability on OHS implementation. Prior research has also emphasized the need to assign responsibilities to employees (46). Employees who feel empowered take responsibility for their safety (72) and hold themselves accountable (46). As a consequence, there is better OHS implementation, which reduces occupational injuries and diseases. Finally, the interaction effect of safety accountability and mimetic motives on OHS implementation was not supported. A possible explanation for this result is that safety accountability is generally perceived as a punitive practice, while mimetic motives encourage organizations to mimic best practices. Consequently, employees resist imitating such practices. Another possible reason might be the infancy stage of OHS implementation in the agriculture sector of Pakistan. OHS implementation is viewed as the responsibility of the OHS department or safety staff only. Therefore, other departments and employees bear no responsibility for safety.

### Theoretical implications

This study has fourfold theoretical implications. First, it adds to the body of OHS literature by combining organizational decision-making styles, safety accountability, and mimetic motives for OHS implementation through the lens of institutional theory. Second, although the importance of safety management practices for OHS implementation has been proven in past studies, these constructs, to the best of our knowledge, have been brought together into a single model for the first time. Third, the organization's style of implementation and safety accountability are important predictors in achieving OHS implementation. However, this study contributes to the sense that the choice of implementation style depends on the organizational competencies and priorities. Finally, previous research such as Iatridis et al. (52) and Hillebrand et al. (58) have examined the interactive effect of mimetic motives in the context of the certified management system and customer relationship management implementation, respectively. This research has looked at its moderating effect in the field of OHS and has discovered that mimetic motives play a significant role in OHS implementation in the Pakistani agriculture sector.

### Practical implications

Overall, the findings of this study have a number of practical implications for OHS professionals, OHS practitioners, and national authorities for better OHS implementation. First, the findings of this study might enable OHS professionals in designing and developing an OHS implementation program by keeping in mind the organizational competencies and priorities. Second, the findings may help OHS practitioners to execute OHS plans and procedures in accordance with the duties and responsibilities that have been assigned to them. Third, it may enable the national authorities in making better policies and decisions about OHS training, field demonstrations, and technology selection for OHS implementation. Fourth, it may help agriculture organizations to be vigilant about safety-related market developments, especially their competitors' best practices. This could help them to stay in business and gain legitimacy in the long run. Finally, the findings provide the liberty to agriculture organizations to choose an implementation style for OHS implementation as per their competencies, environment uncertainties, and their desire to be like other organizations.

## Conclusion

We conceptualized OHS implementation as a positive gateway toward OHS management that is based on the organization's decision-making styles and safety accountability. By doing so, we hope to help OHS professionals and practitioners advance their knowledge about the complex phenomenon of fear of failure in OHS implementation. Thus, OHS implementation should not be viewed as a social or technical issue alone. Instead, arrangements should be made at the organizational level to gain better control over OHS challenges by considering the organizational factors as well as the institutional environment in which the organization operates and to attain legitimacy for survival.

## Limitations and future directions

This research adds new empirical insights to the growing body of literature studies on OHS implementation in the fields of the agriculture sector. There are, however, three major caveats that could be addressed in future studies. First, safety management practices such as management commitment, safety training, and others were not included. Future studies are welcome to measure the impact of safety management practices on OHS implementation in the presence of mimetic motives. Second, the current study was purely cross-sectional in nature which showed a static picture of the OHS phenomenon. Future researchers are encouraged to apply qualitative methods or perform longitudinal studies to examine the dynamic picture of OHS implementation in the agriculture sector. Last, the overprotectiveness of respondents was also observed during data collection. Many farm owners and staff were reluctant to fill out the questionnaire for fear of repercussions from the government and regulatory bodies. Therefore, future studies must take some suitable measures to avoid this problem.

## Data availability statement

The raw data supporting the conclusions of this article will be made available by the authors, without undue reservation.

## Author contributions

MN conceptualized, designed, and wrote the original draft. MN and MH did the data analysis, interpretation, and secured funds. LS supervised, critically reviewed, and edited the manuscript. All authors contributed to the article and approved the submitted version.

## Conflict of interest

The authors declare that the research was conducted in the absence of any commercial or financial relationships that could be construed as a potential conflict of interest.

## Publisher's note

All claims expressed in this article are solely those of the authors and do not necessarily represent those of their affiliated organizations, or those of the publisher, the editors and the reviewers. Any product that may be evaluated in this article, or claim that may be made by its manufacturer, is not guaranteed or endorsed by the publisher.
